# Maternal Hypertension and Adverse Neurodevelopment in a Cohort of Preterm Infants

**DOI:** 10.1001/jamanetworkopen.2025.7788

**Published:** 2025-04-29

**Authors:** Shipra Jain, Ting Ting Fu, Maria E. Barnes-Davis, Rashmi D. Sahay, Shelley R. Ehrlich, Chunyan Liu, Mounira Habli, Nehal A. Parikh

**Affiliations:** 1 The Perinatal Institute, Cincinnati Children’s Hospital Medical Center, Cincinnati, Ohio; 2 University of Cincinnati College of Medicine, Cincinnati, Ohio; 3 Neurodevelopmental Disorders Prevention Center, Cincinnati Children’s Hospital Medical Center, Cincinnati, Ohio; 4 Division of Biostatistics and Epidemiology, Cincinnati Children’s Hospital Medical Center, Cincinnati, Ohio; 5 Department of Environmental and Public Health Sciences, University of Cincinnati College of Medicine, Cincinnati, Ohio; 6 Trihealth Good Samaritan Hospital, Cincinnati, Ohio

## Abstract

**Importance:**

Whether maternal hypertensive disorders of pregnancy (HDP) confer independent neurodevelopmental deficit risks in premature infants is controversial. Previous studies are limited by inadequate confounding variable control and other biases.

**Objective:**

To evaluate the associations between maternal HDP, especially preeclampsia, and neurodevelopmental outcomes of preterm infants at 2 years’ corrected age.

**Design, Setting, and Participants:**

Regional prospective cohort study of 395 preterm infants (≤32 weeks’ gestation) from 5 level III and IV southeast Ohio neonatal intensive care units from September 2016 to November 2019. Data analysis was conducted in August 2022.

**Exposure:**

HDP, defined by maternal diagnosis of chronic or gestational hypertension or preeclampsia during pregnancy.

**Main Outcomes and Measures:**

Structural brain magnetic resonance imaging was performed at term-equivalent age. Neurodevelopment was assessed by Bayley Scales of Infant and Toddler Development (BSID), Third Edition, between 22 and 26 months’ corrected age. Multivariable regression was used to identify the independent association of HDP and preeclampsia on cognitive (primary outcome), language, and motor development, controlling for several confounders. Mediation analyses were performed to understand if the association with HDP was mediated by its association with birth weight or brain abnormalities.

**Results:**

In a cohort of 395 infants, the median (IQR) gestational age was 29.6 (27.6-31.4) weeks, birth weight was 1230 (950-1628) g, and 210 (53.2%) were male. Of these, 170 (43%) were HDP-exposed, of which 104 of 170 (61%) were exposed to preeclampsia. A total of 341 children (87%) completed the BSID. In adjusted analyses, HDP exposure was negatively associated with BSID cognitive scores (−3.69; 95% CI, −6.69 to −0.68; *P* = .02) and language scores (−4.07; 95% CI, −8.03 to −0.11; *P* = .04). Preeclampsia exposure showed similarly negative but greater associations for BSID scores (−4.85; 95% CI, −8.63 to −1.07; *P* = .01 for cognitive and −6.30; 95% CI, −11.49 to −1.09; *P* = .02 for language scores). Mediation analysis revealed that the association between HDP and cognitive scores was partially mediated by its adverse association with brain abnormalities at term-equivalent age (24% of the total effect; −0.82; 95% CI, −1.72 to −0.13; *P* = .02).

**Conclusions and Relevance:**

In this preterm cohort study, maternal HDP was independently associated with adverse cognitive and language development, with accentuated associations observed in preeclampsia-exposed preterm infants, emphasizing the clinical importance of recognizing HDP as a risk, enabling targeted risk management strategies for closer monitoring and aggressive early intervention in affected populations.

## Key Points

**Question:**

What are the independent associations between maternal hypertensive disorders of pregnancy (HDP) and neurodevelopment of premature infants at 2 years’ corrected age?

**Findings:**

In this cohort study of 395 infants, HDP exposure among preterm infants was significantly associated with adverse cognitive and language outcomes at 2 years’ corrected age, with accentuated associations observed in infants exposed to preeclampsia. Mediation analyses revealed that this association was partially mediated by the association between HDP and brain abnormalities on term-equivalent age structural magnetic resonance imaging.

**Meaning:**

These findings suggest that maternal hypertension is an independent factor associated with risk for adverse neurodevelopment at 2 years’ corrected age in preterm infants.

## Teaser

This cohort study evaluates the associations between maternal hypertensive disorders of pregnancy, especially preeclampsia, and neurodevelopmental outcomes of preterm infants at 2 years’ corrected age.

## Introduction

Preeclampsia affects 2% to 5% of all pregnancies^1,2^ and is associated with premature birth and neurodevelopmental deficits in term infants,^3^ likely due to placental ischemia, hypoxia, inflammation, and oxidative stress that disrupt fetal brain development.^4,5,6,7,8,9,10^ Hypertensive disorders of pregnancy (HDP), a broader category encompassing preeclampsia, gestational hypertension, and chronic hypertension existing before pregnancy, impact 5% to 15% of pregnancies,^11^ with rising prevalence in the US.^12^ HDP share similar mechanisms with preeclampsia that can alter brain development^4,5,6,7,8,9,10,13,14^ and may potentially heighten the risk of long-term cognitive, motor, and behavioral disorders in children.^15,16,17^

While HDP exposure is associated with suboptimal neurodevelopmental outcomes in full term–born children,^18,19,20,21,22,23,24,25^ findings in preterm infants vary. Some studies report worse cognitive^24,26,27,28,29,30^ or motor outcomes,^31,32^ while others found no association.^33,34,35^ A few propose a protective role, reporting better neurodevelopmental outcomes in exposed preterm infants compared with their gestational age (GA)–matched controls.^36,37,38,39,40,41^ This inconsistency may stem from a heterogeneous control group where the apparent protective effect of HDP in preterm infants may not truly reflect reduced risk, but rather the high-risk status of the control preterm newborns exposed to other deleterious factors,^31^ like chorioamnionitis, a known risk factor for preterm birth and adverse neurodevelopmental outcomes.^31,41^

Furthermore, published studies have not differentiated between the indirect effect of HDP through its impact on intrauterine growth and its direct pathophysiological effects.^4,5,6,7,8,9,13^ Rather, researchers have inappropriately controlled for birth weight in their analyses.^33,37,41^ Impaired intrauterine growth lies on the causal pathway between HDP and neurodevelopmental outcomes, making it a mediator instead of a confounder. Controlling for mediators results in collider stratification bias, potentially dampening or nullifying the principal association of interest (ie, HDP and infant neurodevelopment).^42,43^

To address these gaps, we investigated the association between maternal HDP and neurodevelopment in preterm infants born at 32 weeks or less. We hypothesized that HDP independently increases the risk of adverse neurodevelopmental outcomes at 2 years’ corrected age and that birth weight mediates rather than confounds this association.

## Methods

### Study Participants

We included 395 preterm infants (≤32 weeks’ GA), from Cincinnati Infant Neurodevelopment Early Prediction Study (CINEPS), a multisite prospective regional cohort. Eligible infants were recruited from 5 level III and IV neonatal intensive care units (NICUs) in greater Cincinnati (September 2016 through November 2019), representing 95% of all births 32 weeks’ GA or less in the region during this period (eFigure in Supplement 1). Exclusions included infants with chromosomal or congenital anomalies affecting the central nervous system, cyanotic heart disease, or poor-quality brain magnetic resonance imaging (MRI) scans (typically from motion artifacts) at term-equivalent age (TEA). The study was approved by the institutional review board of Cincinnati Children’s Hospital Medical Center with written parental consent. This study followed Strengthening the Reporting of Observational Studies in Epidemiology (STROBE) reporting guidelines.

### Variable Definition

We prospectively collected antenatal maternal and postnatal infant characteristics using standardized definitions.^44^ We classified infants as HDP-exposed if their maternal electronic medical record (EMR) documented chronic hypertension or pregnancy-induced hypertension (PIH, defined as gestational hypertension, preeclampsia, or eclampsia) diagnosed by obstetrics per clinical guidelines.^14,45^ We used the standard definition of stage 2 hypertension (blood pressure above 140 mm Hg systolic or 90 mm Hg diastolic before or during the current pregnancy on at least 2 occasions) when no diagnoses were listed.^46^ Antenatal corticosteroid and magnesium therapy were defined as any dose of betamethasone or magnesium sulfate administered before delivery in accordance with established care guidelines.^45,47^ Histological chorioamnionitis was defined as placental inflammatory infiltration on pathological assessment by pathologists.^48,49^ If placental pathology was absent, clinical chorioamnionitis exposure was imputed as yes and left blank otherwise. Socioeconomic status was defined using a social risk score that included family structure, primary caregiver education, primary earner employment status, household income, language spoken at home, and maternal age at birth. This was identical to the 6-component social risk score validated by Roberts et al,^50^ except we replaced occupation of primary earner (not collected) with household income. Scores range from 0 to 12, with higher scores reflecting higher socioeconomic risk.

### MRI Acquisition

We acquired T1-, T2-, and susceptibility-weighted brain MRI scans using a 3-Tesla Philips Ingenia MRI scanner equipped with a 32-channel head coil at TEA during natural sleep, between 39 and 44 weeks’ postmenstrual age.^14,44^ Before imaging, infants were fed, fitted with earplugs (Instaputty, E.A.R. Inc), swaddled in a blanket, and secured in a vacuum immobilization device (MedVac, CFI Medical Solutions) to enhance comfort and minimize movement.

### MR Imaging Assessment

We characterized brain abnormalities in preterm infants using the global brain abnormality score (GBAS), a composite score evaluating brain injuries and disrupted brain development in cerebral white matter, cortical gray matter, deep nuclear gray matter, and cerebellum.^14,51^ GBAS is a validated tool that objectively assesses preterm brain injury and is predictive of neurodevelopment up to age 10 years.^52,53,54,55^ All qualitative and quantitative MRI assessments were conducted by a single neuroradiologist, blinded to maternal and infant clinical history, following established protocols.^14,44,51^

### Neurodevelopment Assessment

Neurodevelopment was assessed by therapists (masked to HDP and the GBAS) trained per the Eunice Kennedy Shriver National Institute of Child Health and Human Development Neonatal Research Network standards using Bayley Scales of Infant and Toddler Development (BSID), Third Edition, between 22 and 26 months corrected age in the NICU High-Risk Follow-up or the Schubert Research Clinic. BSID is a validated and standardized scaled assessment tool to assess development from 1 to 42 months, consisting of various tasks and activities tailored to specific age groups to evaluate cognitive, motor, language, socioemotional, and adaptive behavior.^56^ Each subtest score ranges between 40 and 160, with a mean (SD) of 100 (15). Scores less than 85 are considered below average, and scores less than 70 indicate severe neurodevelopmental impairment.^56^ Our primary outcome was the BSID cognitive composite. Secondary outcomes included the BSID language (composite of expressive and receptive language scores) and motor (composite of fine and gross motor scores) composite scores.

### Statistical Analysis

Baseline characteristics were compared between groups using the *t* test or Wilcoxon rank sum test for continuous variables and χ^2^ or Fisher exact test for categorical variables. We calculated the standardized mean difference (SMD) to assess differences between those lost to follow-up and those not. Univariate and multivariable regression examined the association between HDP and cognitive (primary), language, and motor BSID scores. Subgroup analyses examined the association between PIH (gestational hypertension, preeclampsia, or eclampsia) vs no HDP and preeclampsia (vs no HDP) and BSID outcomes. We further performed multiple imputation analyses to address bias from loss to follow-up.

**Figure 1.**
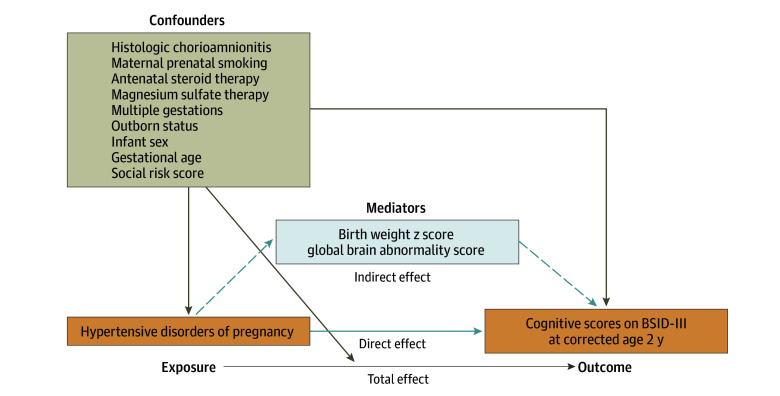
Directed Acyclic Graph Representing Potential Causal Pathways Between Maternal Hypertensive Disorders of Pregnancy, Brain Abnormalities, Confounders, and Neurodevelopmental Outcomes in Preterm Infants This directed acyclic graph represents the causal pathways between maternal hypertensive disorders of pregnancy and preterm infant neurodevelopment at 2 years’ corrected age in preterm infants, illustrating confounders and mediators. BSID-III indicates Bayley Scales of Infant and Toddler Development, Third Edition.

A priori confounders selected based on biological plausibility and previous studies included histologic chorioamnionitis,^48,57^ maternal prenatal smoking (defined as any maternal cigarette smoking during pregnancy),^57,58^ antenatal steroid therapy, magnesium sulfate therapy, multiple gestations, outborn status, infant sex, gestational age, and social risk score. These covariates are associated with our exposure (HDP) and our primary outcome (cognitive development). We did not adjust for neonatal morbidities occurring after the exposure (importantly, birth weight), as we hypothesized that such events lie on the causal pathway between HDP and infant neurodevelopment acting as mediators and partly responsible for later outcomes, thus avoiding collider stratification bias (Figure 1).^43^

We employed mediation analyses^48,57^ using the mediation package in R.^59^ The first analysis distinguished indirect effects of HDP on cognitive BSID scores mediated via poor intrauterine growth (birth weight Fenton *z* scores)^60^ from any potentially direct pathophysiological effects of HDP. The second mediation analysis examined if the association between HDP and infant neurodevelopment was indirectly mediated via early brain injury or abnormal development (GBAS) at TEA. We considered *P* < .05 statistically significant using 2-sided tests. We performed statistical analyses using SAS 9.4 (SAS Institute) and R 4.0.2 (R Project for Statistical Computing) in August 2022.

## Results

In our full cohort of 395 infants, the median (IQR) GA was 29.6 (27.6-31.4) weeks, birth weight was 1230 (950-1628) grams, and 210 (53.2%) were male. Of these, 170 (43%) were HDP-exposed, 134 (34%) were PIH-exposed, and 104 (26%) were preeclampsia-exposed. HDP was diagnosed in 162 mothers via the EMR maternal problem list and 8 through documented blood pressure readings. The HDP and non-HDP groups were comparable, except for maternal age, multiple gestations, histologic chorioamnionitis, and antenatal magnesium therapy (Table 1).^61^ Forty-three percent of infants in the non-HDP group were exposed to histologic chorioamnionitis, compared with 20% in the HDP group. Preeclampsia and non-HDP groups were also comparable except for multiple gestations, histologic chorioamnionitis, antenatal magnesium therapy, and birth hospital (eTable 1 in Supplement 1). PIH and non-HDP groups differed between multiple gestations and histologic chorioamnionitis (eTable 2 in Supplement 1). Two infants withdrew, 1 died, and 341 (87%) completed BSID at 22 to 26 months corrected age, with a median (IQR) follow-up age of 24 (23-26) months. SMDs greater than 0.2 were observed for GA (0.47), birth weight (0.47), social risk score (0.37), and bronchopulmonary dysplasia (0.30) between infants who did and did not attend follow-up (Table 2).

**Table 1.** Prenatal and Postnatal Characteristics of Preterm Infants With and Without Exposure to Hypertensive Disorders of Pregnancy (HDP)

Variable	Infants, No (%)		*P* value
	HDP (n = 170)	Non-HDP (n = 225)	
Maternal characteristics			
Maternal age, mean (SD), y	29.98 (5.60)	28.60 (5.19)	.03
Antenatal steroids	159 (93.53)	203 (90.22)	.24
Antenatal magnesium	151 (88.82)	181 (80.44)	.02
Maternal prenatal smoking	18 (10.59)	32 (14.22)	.28
Histologic chorioamnionitis^a^	33 (20.37)	89 (43.20)	<.01
Social risk score, median (IQR)	3 (1-4)	3 (1-5)	.05
Birth hospital (outborn status)^b^	28 (16.47)	51 (22.66)	.13
Multiple gestations	51(30.00)	91(40.44)	.03
Neonatal characteristics			
Gestational age, median (IQR), wk	30 (28-32)	29 (27-31)	.07
Birth weight, median (IQR), g	1200 (920-1560)	1290 (960-1650)	.26
Birth weight *z* score, mean (SD)	−0.20 (0.94)	0.27 (1.01)	<.01
Sex			
Female	79 (46.47)	106 (47.11)	>.99
Male	91 (53.53)	119 (52.89)	.90
CRIB-2 Score, median (IQR)	6 (2-9)	6 (3-9)	.51
Severe (grade III/IV) intraventricular hemorrhage	9 (5.29)	17 (7.59)	.36
Any intraventricular hemorrhage	38 (22.35)	38 (16.96)	.18
Sepsis	18 (10.59)	26 (11.61)	.75
Necrotizing enterocolitis (stage II or higher)	8 (4.71)	12 (5.36)	.77
Bronchopulmonary dysplasia^c^	66 (39.52)	98 (43.75)	.40
Severe bronchopulmonary dysplasia^c^	27 (15.88)	47 (20.98)	.20
Global brain abnormality scores, median (IQR)	5 (3-8)	4 (2-8)	.21
Outcomes at corrected age, 2 y			
BSID-III Cognitive score, mean (SD)	90.63 (14.45)	90.55 (14.85)	.96
BSID-III Language score, mean (SD)	92.10 (19.84)	90.28 (19.30)	.40
BSID-III Motor score, mean (SD)	93.00 (14.57)	92.14 (14.25)	.59

Abbreviations: CRIB, clinical risk index for babies; BSID-III, Bayley Scales of Infant and Toddler Development, Third Edition.

^a^ As determined by pathologists using Redline^49^ classification. Placental histopathology was available for 364 infants (92%). For missing cases, data were imputed as yes if there was exposure to clinical chorioamnionitis and left blank otherwise.

^b^ Outborn status defined as if the infant was born at a hospital other than the study neonatal intensive care units and needed transfer.

^c^ As defined using the Jenson EA et al^61^ bronchopulmonary dysplasia definition at 36 weeks’ postmenstrual age.

**Table 2.** Prenatal and Postnatal Characteristics of Preterm Infants Who Were Followed Up vs Who Were Lost to Follow-Up

Variables^a^	Follow-up (n = 341)	No follow-up (n = 54)	SMD
Maternal characteristics			
Maternal, mean (SD), y	29.19 (5.41)	29.09 (5.48)	0.02
Antenatal steroids	313 (91.79)	49 (90.74)	0.02
Antenatal magnesium	289 (84.75)	43 (79.63)	0.13
Maternal prenatal smoking	44 (12.9)	6 (11.11)	0.05
Histologic chorioamnionitis^b^	105 (30.79)	17 (31.48)	−0.02
Social risk score, median (IQR)	3 (1-5)	4 (2-5)	−0.37
Birth hospital (outborn status)^c^	69 (20.23)	10 (18.52)	0.04
Multiple gestations	122 (35.78)	20 (37.04)	−0.03
Neonatal characteristics			
Gestational age, wk, median (IQR)	29 (27 − 31)	31(29 − 32)	−0.47
Birth weight, median (IQR), g	1228 (930-1560)	1440 (1108-1780)	−0.47
Birth weight *z* score, mean (SD)	0.06 (0.99)	0.11 (1.11)	−0.05
Sex			
Female	165 (48.39)	20 (37.04)	0.16
Male	176 (51.61)	34 (62.96)	0.23
CRIB-2 score, median (IQR)	6 (3-9)	5 (2-7)	0.19
Severe (grade III/IV) intraventricular hemorrhage	24 (7.06)	2 (3.7)	0.15
Any intraventricular hemorrhage	67 (19.71)	9 (16.67)	0.07
Sepsis	38 (11.18)	6 (11.11)	0.00
Necrotizing enterocolitis (stage II or higher)	17 (5)	3 (5.56)	−0.02
Bronchopulmonary dysplasia^d^	149 (43.82)	15 (29.41)	0.30
Severe bronchopulmonary dysplasia	69 (20.29)	5 (9.26)	0.31

Abbreviations: CRIB, clinical risk index for babies; SMD, standardized mean difference.

^a^ The group difference examined using SMD.

^b^ As determined by pathologists using Redline^49^ classification. Placental histopathology was available for 364 infants (92%). For missing cases, data were imputed as yes if there was exposure to clinical chorioamnionitis and left blank otherwise.

^c^ Outborn status defined as if the infant was born at a hospital other than the study neonatal intensive care units and needed transfer.

^d^ As defined using the Jenson EA et al,^61^ bronchopulmonary dysplasia definition at 36 weeks’ postmenstrual age.

BSID cognitive scores were similar between the groups, with a mean (SD) of 90.63 (14.45) for HDP-exposed and 90.55 (14.85) for unexposed infants (difference, 0.08; 95% CI, −3.52 to 3.57; *P* = .97) (Table 1). GBAS was available for all 395 infants, ranging from 0 to 41 with a median (IQR) of 5 (3-8) in the HDP-exposed group and 4 (2-8) in the unexposed group (difference, 1.00; 95% CI, −1.03 to 0.10; *P* = .21) (Table1). Similar findings were observed in infants exposed to preeclampsia only vs no HDP (eTable 1 in Supplement 1) and PIH vs no HDP (eTable 2 in Supplement 1).

In adjusted models, controlling for a priori confounders, HDP was negatively associated with BSID cognitive (β estimate = −3.69; 95% CI, −6.69 to −0.68; *P* = .02) (Table 3) and language (β estimate = −4.07; 95% CI, −8.03 to −0.11; *P* = .04) (Table 3) scores. Preeclampsia exposure was also associated with lower cognitive (β estimate = −4.85; 95% CI, −8.63 to −1.07; *P* = .01) and language (β estimate = −6.30; 95% CI, −11.49 to −1.09; *P* = .02) scores in adjusted models (Table 3), while PIH was associated with lower cognitive (β estimate = −4.93; 95% CI, −8.32 to −1.55; *P* < .01), language (β estimate = −6.34; 95% CI, −10.92 to −1.77; *P* = .01), and motor (β estimate = −3.62; 95% CI, −7.05 to −0.19; *P* = .04) scores (Table 3). No significant association was found between HDP or preeclampsia exposure and motor scores (Table 3). Imputation analyses accounting for missing BSID outcomes showed similar results (eTable 3 in Supplement 1).

**Table 3.** Association Between Hypertensive Disorders of Pregnancy (HDP), Preeclampsia (PE), and Pregnancy-Induced Hypertension (PIH) With Neurodevelopmental Outcomes at 2 Years’ Corrected Age in Preterm Infants

Exposure and BSID-III outcome	Unadjusted model		Adjusted model^a^	
	β estimate (95% CI)	*P* value	β estimate (95% CI)	*P* value
HDP				
Cognitive	−0.03 (−3.34 to 3.29)	.99	−3.69 (−6.69 to −0.68)	.02
Language	1.41 (−3.02 to 5.85)	.53	−4.07 (−8.03 to −0.11)	.04
Motor	0.78 (−2.40 to 3.96)	.63	−2.81 (−5.84 to 0.22)	.07
PE				
Cognitive	0.18 (−3.65 to 4.01)	.93	−4.85 (−8.63 to −1.07)	.01
Language	2.23 (−3.07 to 7.53)	.41	−6.30 (−11.49 to −1.09)	.02
Motor	2.57 (−1.05 to 6.19)	.16	−2.73 (−6.58 to 1.13)	.16
PIH				
Cognitive	−0.21 (−3.83 to 3.40)	.91	−4.93 (−8.32 to −1.55)	.004
Language	1.26 (−3.65 to 6.17)	.61	−6.34 (−10.92 to −1.77)	.01
Motor	1.27 (−2.18 to 4.72)	.47	−3.62 (−7.05 to −0.19)	.04

Abbreviation: BSID-III, Bayley Scales of Infant and Toddler Development, Third Edition.

^a^ Adjusted for histologic chorioamnionitis, antenatal steroids, magnesium sulfate, maternal prenatal smoking, infant sex, gestational age, multiple gestations, birth hospital, and social risk score.

**Figure 2.**
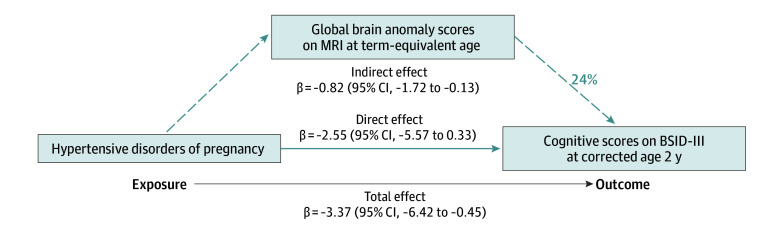
Mediation Model Evaluating the Indirect Effects of Global Brain Abnormality Scores on Magnetic Resonance Imaging (MRI) at Term-Equivalent Age as a Mediator of the Adverse Effects of Hypertensive Disorders of Pregnancy on Cognitive Scores in Preterm Infants This model demonstrates that hypertensive disorders of pregnancy-induced early brain abnormalities are a significant mediator (dashed blue lines) in the pathway between maternal hypertension and lower cognitive scores on the Bayley Scales of Infant and Toddler Development, Third Edition (BSID-III) at 2 years’ corrected age in preterm infants.

In the mediation analysis examining the HDP-induced brain abnormalities on infant neurodevelopment, brain abnormalities partially mediated 24% of the total effect of HDP on lower cognitive scores (β estimate = −0.82; 95% CI, −1.72 to −0.13; *P* = .02) (Figure 2). The remaining majority effect appears to be a direct effect of HDP (β estimate = −2.55; 95% CI, −5.57 to 0.33) on adverse cognitive development but was not statistically significant (*P* = .07) (Figure 2). In mediation analysis, birth weight *z* scores did not show a significant mediation effect on cognitive BSID scores (β estimate = −0.44; 95% CI, −1.26 to 0.20; *P* = .19).

## Discussion

In our regional multisite prospective cohort study, we demonstrated that maternal HDP is adversely associated with cognitive and language development in preterm infants, independent of other perinatal factors. These effects were accentuated in infants born to mothers with PIH or preeclampsia, suggesting that abnormal placentation may be linked to more severe neurodevelopmental impairments in exposed children. Mediation analyses revealed that these effects were partially mediated by aberrant preterm brain development or injury.

Previous studies exploring the impact of HDP on preterm infant neurodevelopment have reported conflicting findings, with some suggesting a protective effect of HDP.^36,37,38,39,40,41^ However, many of these studies suffer from collider stratification bias,^42^ where researchers inappropriately adjusted for intermediate variables such as birth weight^33,41^ that lie on the causal pathway between the exposure (HDP) and outcomes (infant neurodevelopment) and partly mediate the relationship. Adjusting for intermediate variables as confounders diminishes the total effect.^62,63^ For example, Nakamura et al^37^ employed 2 main models, 1 with and 1 without controlling for birth weight, to assess HDP’s effect on preterm infants’ neurodevelopment at age 3 years and revealed that after controlling for birth weight, the odds ratio for adverse development in HDP-exposed infants significantly decreased from 1.34 to 0.93. Our findings of impaired cognitive and language development are consistent with a study that used a similar method and avoided collider bias.^64^ We additionally tested birth weight as a potential mediator of the adverse association between HDP and neurodevelopment but found no significant indirect effect, possibly due to our modest sample size (ie, type II error).

It is important to note that unlike previous studies,^29,35,38,39^ our study controlled for histologic chorioamnionitis, a major risk factor for preterm birth, brain abnormalities,^48^ and neurodevelopmental deficits.^65,66,67^ The perceived protective neurodevelopmental effects of HDP in prior studies likely stemmed from a higher proportion of infants exposed to chorioamnionitis in the comparison control group of preterm infants than in the HDP group, and this difference was not controlled for in the adjusted analysis.^31,39,41^ In our cohort of preterm infants, the non-HDP group was more than twice as likely (43%) to be exposed to histological chorioamnionitis as compared with the HDP group (20%), supporting our rationale to adjust for this confounder in the multivariable model. Studies, including ours, that controlled for chorioamnionitis^37^ or its proxy, such as prolonged rupture of membranes,^32^ consistently reported impaired neurodevelopment in HDP-exposed preterm infants.

In our adjusted models, HDP exposure was associated with a 3.69-point decrease in BSID cognitive scores and a 4.07-point decrease in language scores. Preeclampsia demonstrated greater reductions: −4.85 and −6.30, respectively. A 4- to 6-point mean reduction (approximately one-third SD) in BSID scores can be clinically meaningful, especially at a population level, as slight differences can signal increased risk for later cognitive, language, or motor impairments. Since development is dynamic, even a small early disadvantage can compound over time, especially if the child lacks supportive interventions, potentially affecting school readiness and/or later academic achievement. A one-third SD decrement in cognitive score might not meet intellectual disability thresholds but could increase the likelihood of mild developmental delay in high-risk populations like preterm infants, impacting early interventions and school accommodations (eg, needing specialized services). Furthermore, neurodevelopmental impairments are multifactorial, and no single associated factor will profoundly affect developmental scores. Thus, early identification of subtle differences allows for targeted interventions, such as early speech therapy, occupational therapy, or enriched learning environments, which can improve long-term educational, behavioral, and health outcomes. Our findings align with Chang et al,^68^ where preeclampsia was associated with worse neurodevelopment at age 2 years,^27^ highlighting the critical need for ongoing monitoring and early intervention in HDP-exposed preterm infants, particularly preeclampsia.

We employed mediation analyses to understand the mechanism through which HDP is associated with infants’ neurodevelopment. We found that the HDP’s association with cognitive development was mediated by its adverse effect on early brain development, accounting for 24% of the total effect, rather than fetal growth. We determined brain injury or delayed maturation on TEA brain MRI using the GBAS,^51^ a validated tool in preterm infants that correlates with their later development up to 10 years of age.^52,53,54,55^ We previously demonstrated that HDP-exposed preterm infants had worse GBAS, particularly affecting the white matter.^14^ Our findings thus support an association between HDP and early brain abnormalities and potentially harmful direct effects on cognitive and language development. This may be from factors such as hypoxia-ischemia, oxidative stress, and inflammation, as demonstrated in animal models.^4,5,6,7,8,9,10,13^ Similarly, Su et al^69^ found higher incidence of periventricular leukomalacia in HDP-exposed preterm infants (5.7% vs 1.9%), and Xing et al^70^ observed abnormal white matter development in preterm infants exposed to preeclampsia using diffusion MRI. Consequently, these findings advocate for the incorporation of infant neuroimaging and/or neurodevelopment as key outcomes in future clinical trials evaluating therapies for maternal HDP or preeclampsia.

Recognizing HDP as an independent and substantial risk factor for brain abnormalities on TEA-brain MRI and subsequent compromised neurodevelopment in exposed children offers the potential to refine risk stratification, further aiding in early identification of children at an increased risk of developmental delays, particularly at the time of NICU discharge, using brain MRI findings, thereby assisting in optimal resource allocation. This, in turn, could facilitate closer monitoring and prompt aggressive early intervention support, thus contributing to improved long-term outcomes. Finally, intensified initiatives aimed at preventing or treating maternal hypertension may contribute to reducing the risk of abnormal brain development and neurodevelopmental deficits in preterm infants.

### Strengths and Limitations

Our study has several strengths. We mitigated selection bias by recruiting nearly all eligible preterm infants from 5 regional level III and IV NICUs, encompassing population representation. To ensure reliability, exposure and outcome assessments were conducted with high precision and masking to clinical history. Brain MRI was scored using a standardized scoring system, and BSID-III assessments were performed by trained professionals, thereby reducing measurement error and enhancing result reproducibility. Our research emphasizes the importance of addressing biases arising from incomplete adjustment of confounders (eg, chorioamnionitis) and inappropriate adjustment of intermediate variables such as intrauterine growth that have contributed to the heterogenous infant outcomes following HDP in previous studies. We controlled for key confounders, including histologic chorioamnionitis and socioeconomic status. More than 90% of our samples had placental pathology reports. We used a validated social risk score to assess socioeconomic status. Finally, we used mediation analyses and directed acyclic graphs to reveal previously undemonstrated mechanisms as to how HDP is adversely associated with preterm infant neurodevelopment.

Limitations include the observational nature of our study, including challenges related to establishing causation, potential residual confounding by certain maternal comorbidities, and possible exposure misclassification. HDP diagnosis was derived from maternal EMR as ascertained by the obstetricians, and when no such diagnosis was available, we resorted to the clinical definition of hypertension.^46^ However, only 8 of 170 infants were identified as HDP-exposed using this latter method. We used modified social risk scores due to data constraints. While this adaptation aligns with the validated 6-component score, it may introduce differences between the intended and implemented method. Selection bias may have arisen from our 13% attrition, as infants who return for follow-up are often systematically different from those who do not. However, the no follow-up group had both favorable (larger GA and birth weight and lower bronchopulmonary dysplasia rates) and unfavorable neurodevelopmental risk factors (higher social risk score) that likely canceled each other out. This observation was supported by our multiple imputation sensitivity analyses that showed similar results to the original findings.

## Conclusions

In our regional cohort study of preterm infants, maternal HDP exposure was independently and significantly associated with adverse cognitive and language outcomes at 2 years’ corrected age, with greater effects in PIH or preeclampsia-exposed infants, suggesting that abnormal placentation may lead to more pronounced neurodevelopmental impairments. This association was partly mediated by early brain abnormalities on TEA-MRI, underscoring the importance of further research into the distinct mechanisms through which HDP affects brain development and the development of targeted interventions to support affected infants.

## Notes

Supplement 1. **eTable 1.** Prenatal and postnatal characteristics of preterm infants exposed to preeclampsia versus no exposure to hypertensive disorders of pregnancy **eTable 2.** Prenatal and postnatal characteristics of preterm infants exposed to pregnancy-induced hypertension (PIH) versus no exposure to hypertensive disorders of pregnancy. **eTable 3.** Sensitivity analysis to examine the association between hypertensive disorders of pregnancy (HDP), preeclampsia (PE), and pregnancy-induced hypertension (PIH) with preterm infant neurodevelopment after accounting for lost to follow-up **eFigure.** Flow of participants **eAppendix.** Additional acknowledgements

Supplement 2.
Nonauthor Collaborators

Supplement 3.
Data Sharing Statement
