# Supplementary material for: Maternal Hypertension and Adverse Neurodevelopment in a Cohort of Preterm Infants
Source: JAMA Netw Open. 2025 Apr 29;8(4):e257788. doi: 10.1001/jamanetworkopen.2025.7788 (PMC12042049; doi:10.1001/jamanetworkopen.2025.7788)
Supplement: Supplement 1. — eTable 1. Prenatal and postnatal characteristics of preterm infants exposed to preeclampsia versus no exposure to hypertensive disorders of pregnancy eTable 2. Prenatal and postnatal characteristics of preterm infants exposed to pregnancy-induced hypertension (PIH) versus no exposure to hypertensive disorders of pregnancy. eTable 3. Sensitivity analysis to examine the association between hypertensive disorders of pregnancy (HDP), preeclampsia (PE), and pregnancy-induced hypertension (PIH) with preterm infant neurodevelopment after accounting for lost to follow-up eFigure. Flow of participants eAppendix. Additional acknowledgements [file jamanetwopen-e257788-s001.pdf]

Supplemental Online Content

Jain S, Fu TT, Barnes-Davis ME, et al. Maternal hypertension and adverse neurodevelopment in a cohort of preterm infants. *JAMA Netw Open*. 2025;8(4):e257788.
doi:10.1001/jamanetworkopen.2025.7788

eTable 1. Prenatal and postnatal characteristics of preterm infants exposed to preeclampsia versus no exposure to hypertensive disorders of pregnancy
eTable 2. Prenatal and postnatal characteristics of preterm infants exposed to pregnancy-induced hypertension (PIH) versus no exposure to hypertensive disorders of pregnancy.
eTable 3. Sensitivity analysis to examine the association between hypertensive disorders of pregnancy (HDP), preeclampsia (PE), and pregnancy-induced hypertension (PIH) with preterm infant neurodevelopment after accounting for lost to follow-up
eFigure. Flow of participants
eAppendix. Additional Acknowledgements

This supplemental material has been provided by the authors to give readers additional information about their work.

© 2025 Jain S et al. *JAMA Network Open*.

eTable 1. Prenatal and postnatal characteristics of preterm infants exposed to preeclampsia versus no exposure to hypertensive disorders of pregnancy

Variable[a]	PE(N=104)	Non-HDP (N=225)	P value
**Maternal Characteristics**			
Maternal age in years, mean (SD)	29.38 (5.33)	28.58 (5.19)	0.28
Antenatal steroids	100 (96.15)	203 (90.22)	0.06
Antenatal magnesium	94 (90.38)	181 (80.44)	*0.02*
Maternal prenatal smoking	10 (9.62)	32 (14.22)	0.24
Histologic chorioamnionitis[b]	6 (6.25)	89 (43.2)	*<0.01*
Social risk score, median (q1-q3)	3 (1 - 4)	3 (1 - 5)	
Birth hospital (outborn status)[c]	13 (12.50)	51 (22.66)	*0.03*
Multiple gestations	20 (19.20)	91(40.44)	*<0.01*
**Neonatal Characteristics**			
Gestational age in weeks, median (q1-q3)	30 (29 - 32)	29 (27 - 31)	*0.02*
Birth weight, median (q1-q3)	1229 (945-1641.5)	1290 (960-1650)	0.80
Birth weight Z-score, mean (SD)	-0.34 (0.91)	0.27 (1.01)	<0.01
Male sex	48 (46.15)	119 (52.89)	0.26
CRIB-2 Score, median (q1-q3)	6 (2-9)	6 (3-9)	0.79
Severe (grade III/IV) intraventricular hemorrhage	4 (3.85)	17 (7.59)	0.20
Any intraventricular hemorrhage	21 (20.19)	38 (16.96)	0.48
Sepsis	6 (5.77)	26 (11.61)	0.10
Necrotizing enterocolitis (stage II or higher)	6 (5.77)	12 (5.36)	0.88
Bronchopulmonary dysplasia[d]	36 (34.95)	98 (43.75)	0.13
Severe bronchopulmonary dysplasia	13 (12.5)	47 (20.98)	0.06
Global brain abnormality score, median (q1-q3)	2 (1-4)	2 (1-4)	0.23
**Outcomes at 2 years corrected age**			
Bayley cognitive score, mean (SD)	90.87 (13.24)	90.55 (14.85)	0.73
Bayley language score, mean (SD)	93.04 (20.42)	90.28 (19.30)	0.18
Bayley motor score, mean (SD)	94.74 (13.40)	92.14 (14.25)	0.13

[a]Continuous data presented as mean (SD) and median (q1-q3) and the group difference examined using two sample t-test and Wilcoxon rank sum test as applicable; categorical data presented as count (%) and group difference examined using Chi-Square / Fisher's test.

[b]As determined by pathologists using Redline classification, AJOG. 2015. Placental histopathology was available for 92% of the infants. For missing cases, data were imputed as "yes" if there was exposure to clinical chorioamnionitis and left blank otherwise.

[c]Outborn status defined as if the infant was born at an outside hospital other than the study neonatal intensive care units and needed transfer.

[d]As defined using the Jenson EA et al., 2019 bronchopulmonary dysplasia definition at 36 weeks postmenstrual age.

SD = standard deviation, CRIB=clinical risk index for babies, BSID-III= Bayley Scales of Infant & Toddler Development (BSID), 3rd Edition

© 2025 Jain S et al. *JAMA Network Open*.

eTable 2. Prenatal and postnatal characteristics of preterm infants exposed to pregnancy-induced hypertension (PIH) versus no exposure to hypertensive disorders of pregnancy

Variable[a]	PIH (N=134)	Non-HDP (N=225)	P value
**Maternal Characteristics**			
Maternal age in years, mean (SD)	29.54 (5.36)	28.60 (5.19)	0.09
Antenatal steroids	128 (95.5%)	203 (90.2%)	0.07
Antenatal magnesium	118 (88.1%)	181 (80.4%)	0.06
Maternal prenatal smoking	13 (9.7%)	32 (14.2%)	0.21
Histologic chorioamnionitis[b]	17 (13.5%)	89 (43.2%)	*<0.01*
Social risk score, median (q1-q3)	3 (1 - 4)	3 (1 -5)	0.05
Birth hospital (outborn status[c]	23 (17.2%)	51 (22.7%)	0.21
Multiple gestations	35 (26.1%)	91 (40.4%)	*0.01*
**Neonatal Characteristics**			
Gestational age in weeks, median (q1-q3)	30 (28 - 32)	29 (27 -31)	*0.01*
Birth weight, median (q1-q3)	1208 (940 - 1633)	1290 (960 - 1650)	0.48
Birth weight Z-score, mean (SD)	-0.28 (0.91)	0.27 (1.01)	*<0.01*
Male sex	68 (50.8%)	119 (52.9%)	0.69
CRIB-2 Score, median (q1-q3)	6 (2 - 9)	6 (3 -9)	0.52
Severe (grade III/IV) intraventricular hemorrhage	6 (4.5%)	17 (7.6%)	0.25
Any intraventricular hemorrhage	28 (20.9%)	38 (17%)	0.35
Sepsis	10 (7.5%)	26 (11.6%)	0.21
Necrotizing enterocolitis (stage II or higher)	8 (6%)	12 (5.4%)	0.81
Bronchopulmonary dysplasia[x]	52 (39.1%)	98 (43.8%)	0.39
Severe bronchopulmonary dysplasia[d]	21 (15.7%)	47 (21%)	0.22
Global brain abnormality scores, median (q1-q3)	5 (3 -8)	4 (2 -8)	0.64
**Outcomes at 2 years corrected age**			
BSID-III cognitive score, mean (SD)	90.48 (14.34)	90.55 (14.85)	0.97
BSID-III language score, mean (SD)	92.05 (20.71)	90.28 (19.30)	0.46
BSID-III motor score, mean (SD)	93.41 (14.58)	92.14 (14.25)	0.46

[a]Continuous data presented as mean (SD) and median (q1-q3) and the group difference examined using two sample t-test and Wilcoxon rank sum test as applicable; categorical data presented as count (%) and group difference examined using Chi-Square / Fisher's test.

[b]As determined by pathologists using Redline classification, AJOG. 2015. Placental histopathology was available for 92% of the infants. For missing cases, data were imputed as "yes" if there was exposure to clinical chorioamnionitis and left blank otherwise.

[c]Outborn status defined as if the infant was born at an outside hospital other than the study neonatal intensive care units and needed transfer.

[d]As defined using the Jenson EA et al., 2019 bronchopulmonary dysplasia definition at 36 weeks postmenstrual age.

SD = standard deviation, CRIB=clinical risk index for babies, BSID-III= Bayley Scales of Infant & Toddler Development (BSID), 3rd Edition

© 2025 Jain S et al. *JAMA Network Open*.

eTable 3. Sensitivity analysis to examine the association between hypertensive disorders of pregnancy (HDP), preeclampsia (PE), and pregnancy-induced hypertension (PIH) with preterm infant neurodevelopment after accounting for lost to follow-up

		**Adjusted Model [a]**		**Adjusted Models after doing multiple imputation for missing outcomes**	
**Exposure**	**BSID-III Outcome**	**β-estimate (95% CI)**	**P value**	**β-estimate (95% CI)**	**P value**
HDP	Cognitive	-3.69 (-6.69, -0.68)	*0.02*	-3.50 (-6.34, -0.67)	*0.01*
	Language	-4.07 (-8.03, -0.11)	*0.04*	-3.87 (-7.69, -0.04)	*0.04*
	Motor	-2.81 (-5.84, 0.22)	0.07	-3.04 (-5.91, -0.16)	*0.04*
PE	Cognitive	-4.85 (-8.63, -1.07)	*0.01*	-4.42 (-8.01, -0.84)	*0.01*
	Language	-6.30 (-11.49, -1.09)	*0.02*	-5.29 (-10.32, -0.26)	*0.04*
	Motor	-2.73 (-6.58, 1.13)	0.16	-2.85 (-6.55, 0.84)	0.13
PIH	Cognitive	-4.93 (-8.32, -1.55)	*<0.01*	-4.77 (-7.94, -1.60)	*<0.01*
	Language	-6.34 (-10.92, -1.77)	*0.01*	-5.79 (-10.11, -1.48)	*<0.01*
	Motor	-3.62 (-7.05, -0.19)	*0.04*	-4.01 (-7.39, -0.72)	*0.02*

[a]Adjusted for histologic chorioamnionitis, antenatal steroids, magnesium sulfate, maternal prenatal smoking, infant sex, gestational age, multiple gestations, birth hospital, and social risk score.

HDP= hypertensive disorders of pregnancy, PE = preeclampsia, PIH= pregnancy-induced hypertension, CI = confidence intervals, BSID-III=Bayley Scales of Infant & Toddler Development (BSID), 3rd Edition

© 2025 Jain S et al. *JAMA Network Open*.

eFigure. Flow of participants

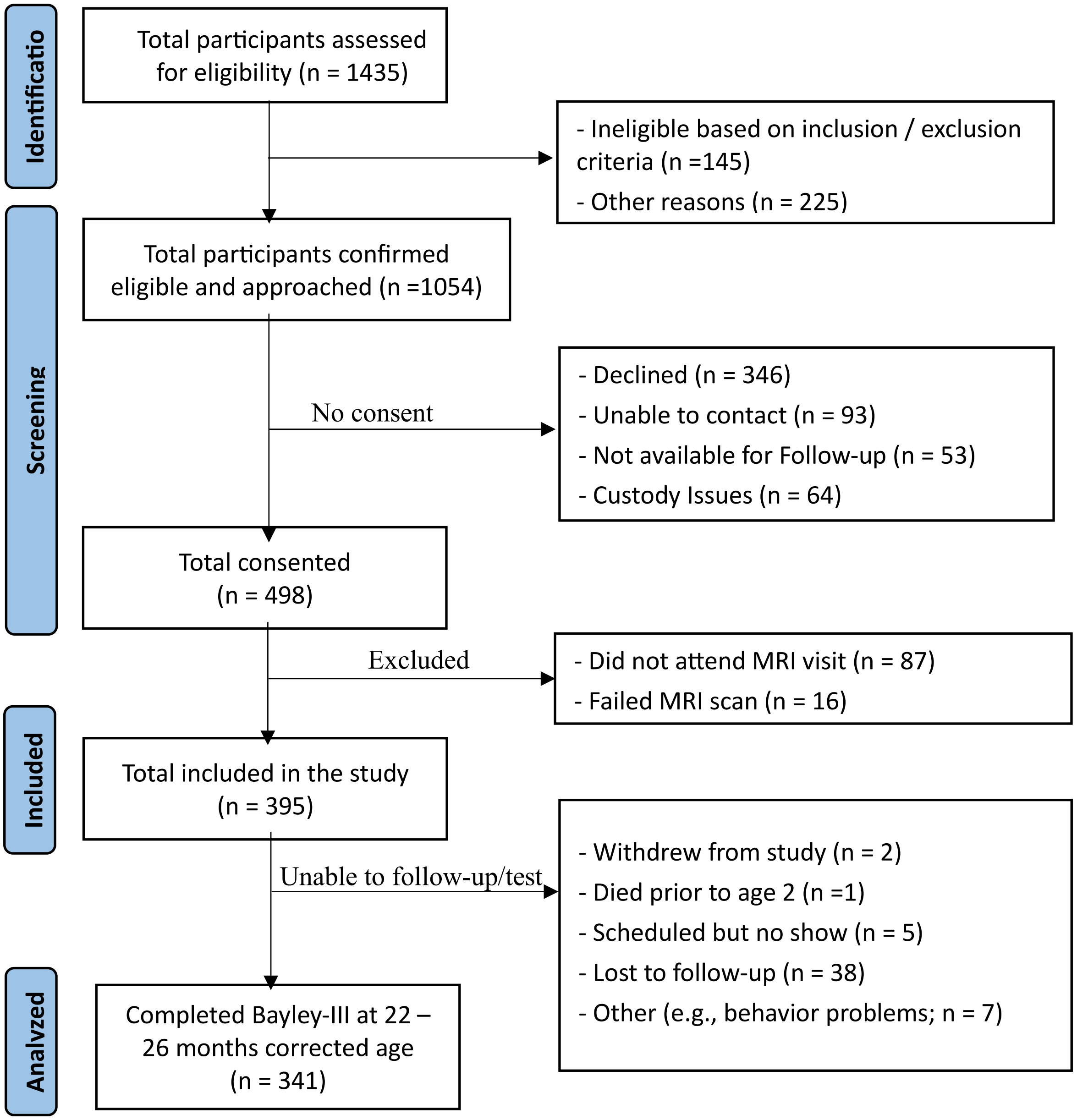

© 2025 Jain S et al. *JAMA Network Open*.

eAppendix. Additional Acknowledgements

We sincerely thank the Cincinnati Infant Neurodevelopment Early Prediction Study (CINEPS) Investigators: Principal Investigator: Nehal A. Parikh, DO, MS. Collaborators (in alphabetical order): Mekibib Altaye, PhD, Anita Arnsperger, RRT, Traci Beiersdorfer, RN BSN, Kaley Bridgewater, RT(MR) CNMT, Tanya Cahill, MD, Kim Cecil, PhD, Kent Dietrich, RT, Christen Distler, BSN RNC-NIC, Juanita Dudley, RN BSN, Brianne Georg, BS, Cathy Grisby, RN BSN CCRC, Lacey Haas, RT(MR) CNMT, Karen Harpster, PhD, OT/RL, Lili He, PhD, Scott K. Holland, PhD, V.S. Priyanka Illapani, MS, Kristin Kirker, CRC, Julia E. Kline, PhD, Beth M. Kline-Fath, MD, Hailong Li, PhD, Matt Lanier, RT(MR) RT(R), Stephanie L. Merhar, MD MS, Greg Muthig, BS, Brenda B. Poindexter, MD MS, David Russell, JD, Sara Stacey, BS, Katie Taylor, BS, Kari Tepe, BSN RNC-NIC, Leanne Tamm, PhD, Julia Thompson, RN BSN, Jean A. Tkach, PhD, Hui Wang, PhD, Hui Wang, PhD, Jinghua Wang, PhD, Brynne Williams, RT(MR) CNMT, Kelsey Wineland, RT(MR) CNMT, Sandra Wuertz, RN BSN CCRP, Donna Wuest, AS, and Weihong Yuan, PhD.

© 2025 Jain S et al. *JAMA Network Open*.
