# Supplementary material for: Maternal Hypertension and Adverse Neurodevelopment in a Cohort of Preterm Infants
Source: JAMA Netw Open. 2025 Apr 29;8(4):e257788. doi: 10.1001/jamanetworkopen.2025.7788 (PMC12042049; doi:10.1001/jamanetworkopen.2025.7788)
Supplement: Supplement 2. — Nonauthor Collaborators [file jamanetwopen-e257788-s002.pdf]

Supplemental Online Content: Nonauthor Collaborators
*First name, last name, and suffix (if applicable) are required and will appear in PubMed.

| *Group Name(s): Cincinnati Infant Neurodevelopment Early Prediction Study (CINEPS) Investigators | | | | | | | |
|---|---|---|---|---|---|---|---|
| ***First Name and Middle Initial(s)** | ***Last Name** | ***Suffix (eg, Jr, III)** | Academic Degrees | Institution | Location (city, state/province, country) | Role or Contribution, eg, chair, principal investigator | Group (if more than 1 Group listed in the byline) and/or Subgroup (eg, Steering Committee) |
| Priyanka VSP | Illapani | | MS | Cincinnati Children's Hospital Medical Center | Cincinnati, OH, USA | Research assistant | |
| Karen | Harpster | | PhD, OTR/L | Cincinnati Children's Hospital Medical Center | Cincinnati, OH, USA | Collaborator | |
| Beth | Kline-Fath | | MD | Cincinnati Children's Hospital Medical Center | Cincinnati, OH, USA | Collaborator | |
| Hailong | Li | | PhD | Cincinnati Children's Hospital Medical Center | Cincinnati, OH, USA | Collaborator | |
| Lili | He | | PhD | Cincinnati Children's Hospital Medical Center | Cincinnati, OH, USA | Collaborator | |
| Stephanie | Merhar | | MD, MS | Cincinnati Children's Hospital Medical Center | Cincinnati, OH, USA | Collaborator | |
| Jean | Tkach | | PhD | Cincinnati Children's Hospital Medical Center | Cincinnati, OH, USA | Collaborator | |
| Hui | Wang | | PhD | Cincinnati Children's Hospital Medical Center | Cincinnati, OH, USA | Collaborator | |
