# Supplementary material for: Maternal Hypertension and Adverse Neurodevelopment in a Cohort of Preterm Infants
Source: JAMA Netw Open. 2025 Apr 29;8(4):e257788. doi: 10.1001/jamanetworkopen.2025.7788 (PMC12042049; doi:10.1001/jamanetworkopen.2025.7788)
Supplement: Supplement 3. — Data Sharing Statement [file jamanetwopen-e257788-s003.pdf]

Data Sharing Statement

Jain. Maternal Hypertension and Adverse Neurodevelopment in a Cohort of Preterm Infants. *JAMA Netw Open*. Published April 29, 2025. doi:10.1001/jamanetworkopen.2025.7788

Data
Data available: Yes
Data types: Deidentified participant data
How to access data: The data will be provided by the corresponding author upon request. Email: Nehal.Parikh@cchmc.org
When available: With publication

Supporting Documents
Document types: None

Additional Information
Who can access the data: anyone requesting the data, researchers whose proposed use of the data has been approved
Types of analyses: for any purpose
Mechanisms of data availability: With a signed data access agreement
